# Uranium Leaching from Contaminated Soil Utilizing Rhamnolipid, EDTA, and Citric Acid

**DOI:** 10.1155/2014/462514

**Published:** 2014-07-22

**Authors:** Sara Asselin, Jani C. Ingram

**Affiliations:** 1 Department of Chemistry and Biochemistry, Northern Arizona University, Flagstaff, AZ, USA; 2 Deerpoint Group, Inc., Fresno, CA, USA

## Abstract

Biosurfactants have recently gained attention as “green” agents that can be used to enhance the remediation of heavy metals and some organic matter in contaminated soils. The overall objective of this paper was to investigate rhamnolipid, a microbial produced biosurfactant, and its ability to leach uranium present in contaminated soil from an abandoned mine site. Soil samples were collected from two locations in northern Arizona: Cameron (site of open pit mining) and Leupp (control—no mining). The approach taken was to first determine the total uranium content in each soil using a hydrofluoric acid digestion, then comparing the amount of metal removed by rhamnolipid to other chelating agents EDTA and citric acid, and finally determining the amount of soluble metal in the soil matrix using a sequential extraction. Results suggested a complex system for metal removal from soil utilizing rhamnolipid. It was determined that rhamnolipid at a concentration of 150 *μ*M was as effective as EDTA but not as effective as citric acid for the removal of soluble uranium. However, the rhamnolipid was only slightly better at removing uranium from the mining soil compared to a purified water control. Overall, this study demonstrated that rhamnolipid ability to remove uranium from contaminated soil is comparable to EDTA and to a lesser extent citric acid, but, for the soils investigated, it is not significantly better than a simple water wash.

## 1. Introduction

The need for uranium in the 1940s through the 1970s spurred the excavating of approximately 1200 uranium mines on the Navajo Reservation [1] in northern Arizona. When the mining ceased, many of the mines were left without sealing tunnel openings, filling open pits, or removing piles of radioactive uranium mine waste. As a result, Navajo miners and local communities have been exposed to elevated levels of uranium and other waste materials [2]. Tailing piles were often left uncovered, resulting in the dispersion of material by wind and rain. Leaching of uranium and other metals may contribute to increased ground and water contamination [3].

The accumulation of toxic metals in soil and aqueous environments has potential health hazards for humans. Because metals do not degrade in the environment in contrast to organic compounds, remediation must involve either immobilization or removal. Cost effective metal removal from aqueous environments has been demonstrated utilizing tree leaves [4], peanut shells [5], crab-shell chitin [6], algal biomass [7], and activated carbon from coconut shell [8].

Metals associated with minerals in the environment may exist as transferrable species that are aqueous or bound to colloids or sediments; their binding is exchangeable through environmental processes such as precipitation and leaching [9]. Alternatively, metals may be tightly bound within the matrix of sediments and are largely unavailable to bacterial and environmental influences making them less mobile in the environment [9]. Because these bound metals are largely immobile based on a geological timescale, their environmental impacts are of less concern. However, metals that are loosely bound to soil are considered environmentally transferrable and are believed to have the greatest impact regarding their transport and bioavailability.

The use of chemicals such as ethylenediaminetetraacetic acid (EDTA) has been extensively studied as a metal chelating agent. Zinc, cadmium, copper, and lead were shown to extract 1: 1 in metal-EDTA complexes from contaminated soils [10]. EDDS (ethylenediaminedisuccinic acid) has also been shown to enhance solubility of uranium in contaminated soils [11]. Phosphorus sources such as bone meal, which is primarily calcium phosphate, have been shown to form insoluble metal-phosphate complexes to reduce metal release in soils [12]. Another approach is the addition of phosphoric acid to combine with phosphate rock to form insoluble phosphorus-containing minerals to immobilize the metal in the environment [13]. Soil and sediment remediation have been demonstrated using coal fly ash [14]. Citric acid and sodium bicarbonate were shown to remove 20 to 60% of depleted uranium from contaminated soil [15]. It was also shown that the presence of carbonates in soil may reduce leachability of uranium and other heavy metals due to the buffering capacity of the carbonate [16].

Microbial products are becoming more popular as a green alternative to synthetic chemical techniques. Rhamnolipids are glycolipids produced by the bacteria *Pseudomonas aeruginosa* and are gaining attention as an effective agent to complex metals such as lead, cadmium, and zinc [17]. They have been shown to facilitate the removal of heavy metals from soil, water, and other contaminated surfaces. Rhamnolipids come in predominant two forms, monorhamnolipid and dirhamnolipid, differing by the number of rhamnose sugars. Monorhamnolipid is thought to be more efficient at metal removal than dirhamnolipid [18]. The rhamnolipid harvested from the bacteria is a mixture of four different monorhamnolipids differing by the number of carbons on the fatty chain and has an average molecular weight of 504 g/mol [19]. A study performed by Wen compared the degradation rates of rhamnolipid to citric acid and EDTA. This study found that 20% of the citric acid in the soil was degraded in four days, while 70% was degraded in 20 days. EDTA was much more persistent with only 14% degraded after 20 days. Rhamnolipid had a degradation rate in between citric acid and EDTA, suggesting it would persist in the soil long enough to have an effect on metal removal [20].

The goal of this work was to determine the efficiency of rhamnolipid to aid in the removal of soluble uranium from contaminated soil collected from the Navajo Reservation. This was performed by first determining the total uranium content in the soil using a hydrofluoric acid digestion, then comparing the amount of metal removed by rhamnolipid to other chelating agents such as EDTA and citric acid, and finally determining the amount of soluble metal in the soil matrix using a sequential extraction.

## 2. Methods

### 2.1. Field Work

Soil samples were collected from two locations on the Navajo Reservation in Arizona: Cameron and Leupp. Global positioning coordinates (GPS) for the locations are in Table 1. Open pit uranium mining occurred in and around the community of Cameron, Arizona. As a control site, Leupp, Arizona, was sampled because no mining took place in this community also located on the Navajo Reservation. The two sites are approximately 45 miles apart. Soil collected from the Cameron area has approximately two to four times higher uranium levels compared to soil from Leupp, which is at natural background levels of approximately 3 *μ*g/g [21]. The soil collected from Cameron will be referred to as mining soil, while the soil collected from Leupp will be referred to as the control soil. Topsoil no deeper than 30 cm (collected twice from each site) was shoveled into large plastic tubs and returned to the laboratory. Approximately, 10 kg of soil was collected from each site. The soil was air dried by spreading a thin layer (approximately 0.5 cm height) of soil on paper plates and covering with large paper towels (KimWipes) for two days before any further work was done. A ball mill (Spex 8000M fitted with tungsten carbide grinding balls and tungsten carbide vial) was used to crush the soil samples, and a series of sieves (VWR) were used with pore sizes ranging from 1mm to 70 *μ*m. The fraction of the soil above 70 *μ*m was not used in the study.

### 2.2. Acid Dissolution

Dried soil collected from the mining and control sites was prepared as described in Section 2.1. The crushed soil sieved to less than 70 *μ*m (VWR) was transferred to 2 mL amber glass vials (Wheaton Lot number 224981) and filled approximately half full. Samples were then ashed at 550°C for 24 hours. Approximately, 0.2 g of the ashed material was weighed into a 50 mL polypropylene centrifuge tube (VWRLot #186415), and 2.5 mL of concentrated HNO_3_ (BDH Lot #11110510) and 1.5 mL of concentrated HF (BDH5210020) were added. The reaction rate was increased by placing the samples in an 80°C oven overnight. An aliquot of 0.77 g ± 0.02 g granular boric acid (BDH Lot #82312) was added to neutralize the HF and then adjusted to a total volume of 50mL with purified water (18 MΩ · cm). The sample was then heated on high (1000 Watts) in a microwave oven (Magic Chef) for two minutes and then allowed to cool. The end result was a complete dissolution of the soil sample; this was assumed due to the lack of any visible soil residue. The sample was diluted by a factor of 50 by mixing 0.1 mL of the sample with a dilution mix containing U^233^ as an internal standard prepared from a stock solution of IRMM-058, obtained from the Institute for Reference Materials and Measurements (Geel, Belgium), 1% HNO_3_, and purified water (18 MΩ · cm). Samples were then analyzed using a Thermo X Series 2 ICP-MS for metal content using calibration standards prepared with U^238^ (CPI International Lot number 09J031) ranging from 0 to 1 ppb. The acid digestion was also performed on certified NIST standard reference material SRM 2709 and SRM 2710 soil to verify complete metal removal from the soil using this method.

### 2.3. Seventy-Two hr Leaching with Rhamnolipid, EDTA, and Citric Acid

To investigate the effect rhamnolipid, EDTA, and citric acid had on soluble metal leaching, various concentrations of each chelating agent were used. The microbial form of rhamnolipid was obtained from Dr. Raina Maier (University of Arizona, Tucson, AZ) where it was harvested from bacteria and purified [22]. The bacteria used for this study were Pseudomonas aeruginosa (ATCC 9027) which is known to predominantly produce monorhamnolipid [23]. The monorhamnolipid was prepared at concentrations of 10, 50, 150, and 300 *μ*M, and the EDTA and citric acid were prepared at concentrations of 150 *μ*M and 2.5 mM. Each solution was prepared using purified water (18 MΩ · cm). Henceforth, the monorhamnolipid will be referred to simply as rhamnolipid.

The dried and sieved soil (0.25 g) was weighed into 10 mL polypropylene transport tubes (VWR Lot number 102235) and 10 mL of the prepared chelant solution was added to each sample. The solution was placed in a test tube rack, turned on its side, and then secured on an orbital shaker table (Thermo MaxQ 3000) for 72 hrs. The samples were then filtered using disposable 0.45 *μ*m filters (Whatman Lot #Z532) with disposable syringes (National Scientific Lot #00113327). Each sample was prepared in triplicate along with procedural blanks and repeated three times to ensure reproducibility of results. A small amount of the sample (0.1 mL) was then diluted to a total volume of 10 mL with a dilution mix containing the internal standard U^233^, 1% HNO_3_, and purified water (18 MΩ · cm). Samples were analyzed for trace metal content using the Thermo X Series 2 ICP-MS with calibration standards prepared with U^238^ ranging from 0 to 1 ppb.

### 2.4. Sequential Extraction

The seven-step sequential extraction per Quejido [24] sequentially leaches water soluble salts, exchangeable cations, carbonates, HCl soluble compounds, oxidizable phases, and finally insoluble residues. This was performed after a rhamnolipid extraction to determine not only the amount of soluble metal removed by the rhamnolipid but the speciation of the soluble metal removed in the soil after rhamnolipid treatment (Table 2) as well. Dried soil collected from the mining and control sites was prepared as described in Section 2.1. A total of 60 samples were prepared, 30 containing the soil from the mining area and 30 containing the soil from control area in addition to procedural blanks. This allowed for each sample to be performed in triplicate during a single experiment as well as repeating the experiment three times.

The initial treatment of the soils was a 72 hr leaching of the soil with a rhamnolipid solution. Twenty mL of the rhamnolipid solution was added to each soil sample and stirred for three days on a stir plate using a Teflon stir bar. The samples were then centrifuged (Jouan CR3i) at 6000 rpm for 30 minutes. The supernatant was removed and saved for analysis, and the soil residue was saved for the sequential extraction. Next, the same soil underwent a seven-step sequential extraction. Each step is described in Table 2. Note that, at the end of each step, the soil was centrifuged to enable separation of the extract for analysis.

Each sample extract was filtered with a 0.45 *μ*m disposable filter and disposable syringe and diluted 1: 100 with a dilution mix containing U^233^ as an internal standard, 1% HNO^3^, and purified water (18 MΩ · cm). Samples were analyzed for trace metal content using a Thermo X series 2 ICP-MS with calibration standards prepared with U^238^ ranging from 0 to 1 ppb.

## 3. Results and Discussion

### 3.1. Acid Dissolution

An ICP-MS was used to determine the uranium content of the soil samples collected from mining and control sites and certified NIST SRM soils, which underwent a hydrofluoric acid digestion with nitric acid and boric acid (Section 2.2). Soil samples were collected twice during this project from the same approximate location. Physical appearances of the soils from the two sites were different. The soils were chosen for this study based on proximity to mining activity as well as location on the Navajo Reservation. Although the comparison of these soils is not ideal due to the differences, the soils do represent potential remediation sites of interest. The control soil was dark red in color and sandy in texture, while the soil from mining site was gray in color and was rich in clay. The clayminerals in soil were determined by X-ray diffraction at the University of Arizona. The mining soil contained kaolinite and smectite as well as quartz; control soil also contained kaolinite but had mica/illite with small amount of vermiculite. The HF acid digestion determined the uranium concentrations to be approximately 5 *μ*g/g in the mining soil and 2 *μ*g/g in the control soil (Table 3). The units, *μ*g/g, refer to *μ*g of uranium per gram of dried soil. The difference in metal content between the 2010 and 2011 collection could be explained by the lack of homogeneity of the soil samples. The acid digestion method was assumed to solubilize the metals present in the soil samples by complete digestion of metals. Two certified soils, NIST SRM 2709 and NIST SRM 2710, were digested to verify the acid digestion method. The noncertified value for uranium in NIST SRM 2709 was 3 *μ*g/g; the acid digestion method result was determined to be 2.8 ± 0.06 *μ*g/g in the sample. The noncertified value for uranium in NIST SRM 2710 was 25 *μ*g/g, and the acid digestion determined 28.4 ± 0.5 *μ*g/g in the sample. These results were deemed satisfactory as verification of the acid digestion method.

### 3.2. Seventy-Two hr Leaching with Rhamnolipid, EDTA and Citric Acid

**Figure 1:**
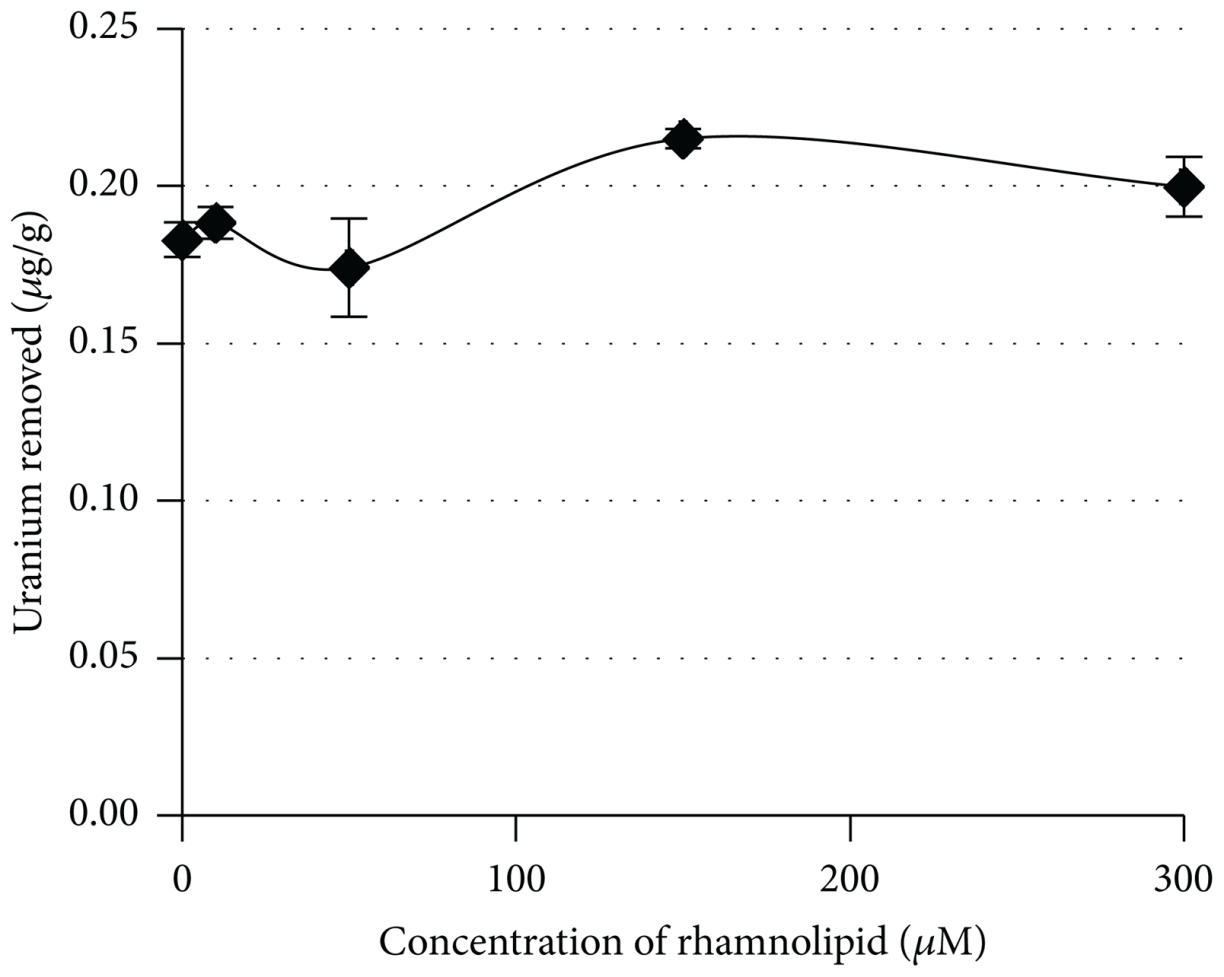
Removal of uranium as a function of the concentration of microbial rhamnolipid in the solution is shown.

**Figure 2:**
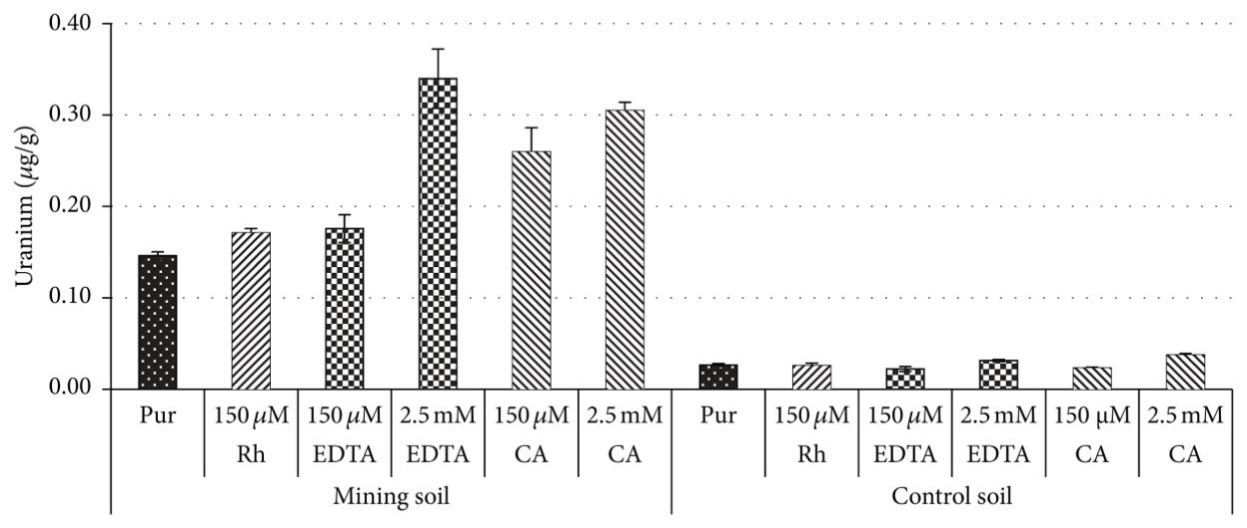
Removal of uranium from the mining and control soils utilizing rhamnolipid (150 *μ*M), EDTA (150 *μ*M and 2.5 mM), and citric acid (150 *μ*M and 2.5 mM). Pur: purified water (18 MΩ · cm), Rh: rhamnolipid, and CA: citric acid.

Studies by Pemberton [25] suggest that the optimum soluble metal removal is near the critical micelle concentration (CMC). Their work suggests that the CMC is pH dependent. They reported that at pH 4 the CMC was 10 *μ*M, at pH 6 the CMC was 100 *μ*M, and at a pH 8 the CMC was 190 *μ*M. Based on the known soil pH of 7.5, it was predicted that the optimum concentration for soluble metal removal would be between 100 and 200 *μ*M. This prediction was confirmed and determined to be near 150 *μ*M by testing various concentrations of rhamnolipid (0–300 *μ*M) and measuring the amount of soluble metal removed (Figure 1). Using the 150 *μ*M rhamnolipid, it was compared to the chelating ability of EDTA and citric acid. EDTA and citric acid were prepared at concentrations of 150 *μ*M and 2.5mM. It was determined that for the mining soil, rhamnolipid and EDTA at 150 *μ*M concentrations had similar chelating effects at 0.17 and 0.18 ug/g, respectively. These results are somewhat better than uranium removal by simple water washing which gave 0.15 *μ*g/g results. The citric acid performed better than either the rhamnolipid or the EDTA at 0.26 *μ*g/g for the mining soil which is approximately a factor of 50% improvement. At a concentration of 2.5 mM, EDTA and citric acid outperformed 150 *μ*M rhamnolipid (Figure 2). The removal of uranium by all treatments (water, rhamnolipid, EDTA, and citric acid) was minimal.

### 3.3. Sequential Extraction

The objective of this experiment was to investigate the hypothesis that soluble metal removal was dependent upon the speciation of the metal in the soil matrix, specifically uranium that is transportable in the environment as a carbonate or water soluble species. The approach used in this experiment was a low-cost, controlled, sequential extraction that selectively removed components from the soil as a function of the solvent selected. This was done after the soil had been exposed to either a 72 hr rhamnolipid extraction or 72 hr water only (control) extraction. By performing the experiment in this way, the species of soluble metal removed by the rhamnolipid could be determined by comparing soil exposed to rhamnolipid and the control. The major assumption was that any difference in metal content was a result of the rhamnolipid solution.

**Figure 3:**
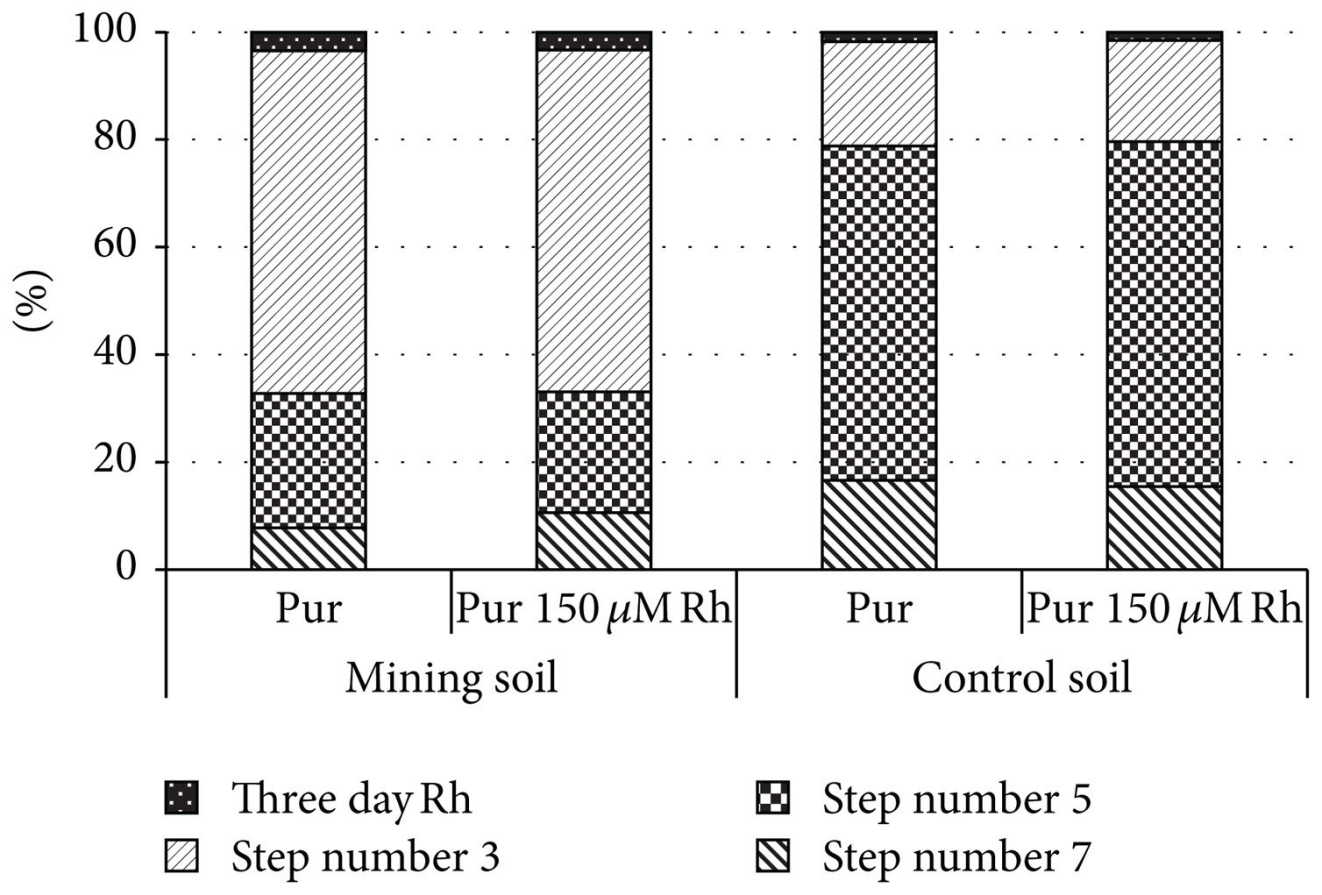
The percent uranium removed from each of the four relevant steps of the sequential extraction was compared (see Table 2). The speciation of the uranium could be determined by comparing the rhamnolipid solutions to the corresponding water only solution.

It was determined that only three of the seven sequential extraction steps removed any uranium from the soil (Steps 3, 5, and 7), and it was confirmed that the sequential extraction method did remove all the uranium from the soil. This was established by summing the uranium removed from the relevant sequential extraction steps and comparing the sum to the value determined by the HF acid digestion. The sequential extraction experiment determined that greater than 50% of uranium in the mining soil was in either a carbonate or phosphate bound species, and less than 25% of the uranium in the control soil was in either a carbonate or phosphate bound species (Figure 3). This would explain why a greater percentage of soluble uranium was removed from the mining soil than the control soil using rhamnolipid. These results indicated how the metal binding to the soil directly affects the rhamnolipid’s ability to remove it from the soil matrix. These results confirmed the hypothesis that metal removal was dependent on the speciation of the metals bound within the soil matrix.

## 4. Conclusion

It was determined that, at a concentration of 150 *μ*M, rhamnolipid was as effective as EDTA for the removal of soluble uranium from the mining soil. However, the rhamnolipid result was only slightly better than the result observed for the purified water control. The citric acid leaching was markedly better than the rhamnolipid at removing uranium from the mining soil. When the concentration of EDTA and citric acid was increased to 2.5 mM, the amount of soluble uranium removed from the mining soil increased. It was also observed that the species of uranium is important for removal. The mining soil was determined to have a much larger percentage of soluble uranium in the soil; therefore, the chelant was able to remove a larger percentage of uranium. Future studies should focus on the pH affects with respect to effectiveness. With further research, it is predicted that rhamnolipid does have the ability to be a useful green alternative for soluble metal removal from contaminated soils.

## Figures and Tables

**Table 1:** GPS coordinates for soil collection sites. This is a summary of the soil collection sites and the corresponding GPS coordinates.

	GPS latitude	GPS longitude
Mining (Cameron) 2010	N 35° 51′ 10.8″	W 111° 25′ 43.2″
Mining (Cameron)2011	N 35° 54′ 48.4″	W 111° 23′ 44.0″
Control (Leupp) 2010	N 35° 17′ 48.1″	W 110° 59′ 53.2″
Control (Leupp) 2011	N 35° 14′ 40.5″	W 111° 01′ 1.8″

**Table 2:** The procedure followed for the sequential extraction paired with the rhamnolipid extraction. The table includes the solvent utilized and the duration and temperature of the agitation for the extraction.

Step	Solvent	Duration/temperature
	Rhamnolipid extraction	Seventy-two hr at room temp
1	Subboiling purified water (20 mL)	1 hr at 95°C.
2	1 M ammonium chloride (8 mL)	1 hr at room temp.
3	1 M ammonium acetate (20 mL) at pH	4 hr in 85°C hot water bath
4	Mixture of 10.9 g/mL oxalic acid and 16.2 g/L ammonium oxalate (20 mL)	4 hr at room temp. in the dark
5	Six M hydrochloric acid (30 mL)	2 hr at 85°C hot water bath
6	(a) 8.8 M hydrogen peroxide (5 mL, pH 2) (b) 8.8 M hydrogen peroxide (5 mL, pH2) (c) 1 M ammonium acetate (25 mL, pH 4)	(a) room temperature for 1 hr uncovered, then 85°C uncovered until volume reduced to 2 ml (b) 85°C to reduce to 2 mL (c) room temperature, covered, and stirred for 18 hr
7	(a) hydrofluoric acid/aqua regia (5 mL) (b) perchloric acid (5 mL)	(a) 30 min. room temp (b) 10 min room temp

Chemicals: ammonium chloride (BDH Lot number 78688), ammonium acetate (OmniPur Lot number TF07DZEMS), oxalic acid (EMD Lot number TD30EZEMS), ammonium oxalate (J.T. Baker Lot number G46147), hydrochloric acid (BDH Lot number 87003-216), hydrogen peroxide (BDH Lot number 107402), and perchloric acid (BDH Lot number 142568).

**Table 3:** The average metal found of the four soil samples (*μ*g/g), along with the limit of detection for each metal.

	Mining		Control		LOD (ppb)
	Cameron ′10	Cameron ′11	Leupp ′10	Leupp ′11	
	*μ*g/g		*μ*g/g		*μ*g/g
**238U**	**6.5**	**4.1**	**2.0**	**1.4**	**0.018**
St. Dev	0.2	0.1	0.1	0.02	
